# Evaluating the Psychometric Properties of a Physical Activity and Sedentary Behavior Identity Scale: Survey Study With Two Independent Samples of Adults in the United States

**DOI:** 10.2196/59950

**Published:** 2024-10-24

**Authors:** Cheng K Fred Wen, Stefan Schneider, Doerte U Junghaenel, Meynard John L Toledo, Pey-Jiuan Lee, Joshua M Smyth, Arthur A Stone

**Affiliations:** 1 Dornsife Center for Self-Report Science University of Southern California Los Angeles, CA United States; 2 Department of Psychology University of Southern California Los Angeles, CA United States; 3 Department of Psychology The Ohio State University Columbus, OH United States

**Keywords:** physical activity, sedentary behavior, geriatrics, exercise, lifestyle, physical health, mental health, social-cognitive approach

## Abstract

**Background:**

Emerging evidence suggests a positive association between relevant aspects of one’s psychological identity and physical activity engagement, but the current understanding of this relationship is primarily based on scales designed to assess identity as a person who exercises, leaving out essential aspects of physical activities (eg, incidental and occupational physical activity) and sedentary behavior.

**Objective:**

The goal of this study is to evaluate the validity of a new physical activity and sedentary behavior (PA/SB) identity scale using 2 independent samples of US adults.

**Methods:**

In study 1, participants answered 21 candidate items for the PA/SB identity scale and completed the International Physical Activity Questionnaire-Short Form (IPAQ-SF). Study 2 participants completed the same PA/SB identity items twice over a 1-week interval and completed the IPAQ-SF at the end. We performed factor analyses to evaluate the structure of the PA/SB identity scale, evaluated convergent validity and test-retest reliability (in study 2) of the final scale scores, and examined their discriminant validity using tests for differences in dependent correlations.

**Results:**

The final PA/SB identity measure was comprised of 3 scales: physical activity role identity (F1), physical activity belief (F2), and sedentary behavior role identity (F3). The scales had high test-retest reliability (Pearson correlation coefficient: F1, *r*=0.87; F2, *r*=0.75; F3, *r*=0.84; intraclass correlation coefficient [ICC]: F1: ICC=0.85; F2: ICC=0.75; F3: ICC=0.84). F1 and F2 were positively correlated with each other (study 1, *r*=0.76; study 2, *r*=0.69), while both were negatively correlated with F3 (Pearson correlation coefficient between F1 and F3: *r*=–0.58 for study 1 and *r*=–0.73 for study 2; F2 and F3: *r*=–0.46 for studies 1 and 2). Data from both studies also demonstrated adequate discriminant validity of the scale developed. Significantly larger correlations with time in vigorous and moderate activities and time walking and sitting assessed by IPAQ-SF with F1, compared with F2, were observed. Significantly larger correlations with time in vigorous and moderate activities with F1, compared with F3, were also observed. Similarly, a larger correlation with time in vigorous activities and a smaller correlation with time walking were observed with F2, compared with F3.

**Conclusions:**

This study provided initial empirical evidence from 2 independent studies on the reliability and validity of the PA/SB identity scales for adults.

## Introduction

The physical and mental health benefits of being physically active [1,2] are well documented. Despite this, however, the vast majority of adults in the United States are insufficiently active [3] and largely sedentary [4]. Although existing physical activity promotion efforts that focus on skill development, barrier reduction, and other important aspects of behavior change have been developed to remedy this, the effects of these interventions have been modest [5,6]. Identifying concepts and approaches beyond current behavioral models could potentially enrich future physical activity promotion and sedentary behavior reduction efforts.

The concept of identity has received increasing attention for its potential to supplement the predominant social-cognitive approaches used in physical activity promotion research [7]. Identity broadly refers to how a person views themselves in a given role (eg, as an exerciser [8]); it is often posited to serve a critical role in regulating behaviors in that people will generally strive to act in a manner that is consistent with their perceived identity [9]. The dissonance experienced when a person deviates from roles they identify with (identity-behavior discordance) could be important motivation for individuals to engage in the target behavior. In the context of promoting physical activity and reducing sedentary behavior, the current literature suggests that individuals with stronger exerciser identity are more physically active [7], and emerging evidence indicates that exerciser identity could predict time spent in exercise in the future [7,10]. The current understanding regarding the relationship between identity and physical activity is largely based on measurement tools such as the Exercise Identity Scale [8]. The Exercise Identity Scale consists of 9 items with questions like “I consider myself an exerciser” “and “I would feel a real loss if I were forced to give up exercising.” Although the Exerciser Identity Scale has been shown to be a psychometrically sound self-report instrument, its focus on exercise is intentionally narrow and does not focus on physical activity more generally or encompass sedentary behaviors at all. According to the “specificity matching principle” [11], the breadth of the concept captured with an identity scale should match the breadth of the behavior it is thought to regulate. The concept of “physical activity” is often construed to include a larger spectrum of activity (eg, inclusive of incidental physical activities like yardwork or occupational physical activities like walking and lifting for work-related activities) than exercise, which refers to a smaller collection of activities that often require planning and involve repetition (eg, running on treadmills, engaging in sports), and can provide robust health benefits [3]. Another limitation is that sedentary behavior is increasingly recognized as a unique and key target for promoting health [3], but self-views about being sedentary are not addressed in the Exercise Identity Scale. Therefore, suitable measurement instruments that assess identities related to physical activity and sedentary behavior may be important for better characterizing and understanding their role in the context of physical activity promotion and sedentary behavior reduction efforts.

To explicitly assess physical activity identity, a small number of studies have modified the wording of the scale from “exercise” to “physical activity” [12,13]. A limitation of these prior studies is that they were conducted with generally small samples, often of older participants, and the psychometric properties of the modified scale for adults in a broad age range were not reported. Additionally, these modified scales did not include the concept of sedentary behavior identity, even though understanding factors that contribute to prolonged engagement in sedentary behavior could potentially offer valuable targets for behavior intervention. To address these limitations, this article evaluated the psychometric properties of a modified version of the Exercise Identity Scale for the purposes of assessing both physical activity identity and sedentary behavior identity. The first aims of the study were to (1) demonstrate the internal consistency of the scales in 2 separate and independent samples and (2) evaluate the test-retest reliability by administering the scale twice to the second sample, with a 7-day interval between administrations. The second aim was to examine the criterion validity of the physical activity and sedentary behavior identity scale by examining the correlations between the scale scores and participants’ self-reported physical activity assessed with the widely used short form of the International Physical Activity Questionnaire (IPAQ-SF) [14].

## Methods

### Measurement

#### Physical Activity and Sedentary Behavior Identity Scale

The physical activity and sedentary behavior identity scale is modified from the Exercise Identity Scale [8]. We included all 9 items from the original Exercise Identity Scale and modified the wording “exercise” or “exerciser” to “physical activity” or “physically active” where relevant. In addition, we also included 3 new items that were intended to assess whether the individual would describe themselves as physically active, how they place themselves in comparison with other people, and how much they enjoy physical activity engagement during leisure time. To assess sedentary behavior identity, items were newly developed with wording paralleling the physical activity identity items. Not all candidate items from the physical activity identity scale were converted into the sedentary behavior identity scale (a total of 9 sedentary items were created), because the contents tapped by several items were seen as not appropriate for sedentary behavior. The list of candidate items for the physical activity and sedentary behavior identity scale is presented in Multimedia Appendix 1.

Participants were asked to rate how strongly they disagreed or agreed with each of the 21 statements on a 7-point Likert scale, with “Strongly Disagree” and “Strongly Agree” anchored at the ends of the scale and a “Neither Disagree Nor Agree” option presented in the middle of the scale. Response options were presented horizontally.

#### Self-Reported Physical Activity and Sedentary Behavior

Levels of physical activity over the past week were assessed using the IPAQ-SF. The IPAQ-SF is a well-validated self-report instrument for assessing physical activity and sedentary behavior during the past 7 days in youth and adults 16 years to 69 years of age [14]. The total times spent in vigorous physical activity, moderate physical activity, and walking during the past 7 days were assessed with 2 questions for each activity category. Participants were asked to recall the number of days they engaged in each activity category. If the participant indicated that they engaged in vigorous physical activity, moderate physical activity, or walking for 1 or more days during the past 7 days, they were then asked to estimate the amount of time they usually spent doing those activities on 1 of those days. The total amount of time spent in each category during the past 7 days was obtained by multiplying the responses of the 2 responses for each category. Sedentary behaviors were assessed using 1 item that asked participants to estimate how much time they spent sitting on a weekday. For all activity categories, participants were also provided with an option to say “Don’t know/Not sure” when they were asked to estimate the time spent in these activities.

### Participants and Procedures

#### Study 1

A total of 1000 participants were recruited through Amazon Mechanical Turk (MTurk) in November 2021. The study invitation was only available to registered MTurk workers (MTurkers) who had already completed a minimum of 500 approved human intelligence tasks (HITs), had at least a 99% HIT approval rate, and lived in the continental United States. Two additional eligibility criteria for study participation included being at least 18 years of age and having English as the first language. Eligible MTurkers were presented with a link to a Qualtrics survey and were asked to only complete the survey using a desktop, laptop, or tablet. Surveys completed on a smartphone were not accepted to ensure that the presentation of survey questions was consistent for all respondents (eg, surveys presented on smartphones using smaller fonts on a smaller screen may force response options to be presented in substantially altered ways, such as vertically, and this may have altered results).

The study survey began with questions about demographic and socioeconomic status, then the participants were presented a paragraph defining physical activity, differentiating exercise from physical activity, and defining sedentary behavior to minimize the potential impact of individual differences in the definition of physical activity, sedentary behavior, and exercise. The definitions of physical activity and sedentary behavior were as follows: “Physical activity is defined as any movement you do with your muscles that requires your body to use energy. The term ‘physical activity’ should not be mistaken for ‘exercise.’ Exercise is only one type of physical activity that you do; it is oftentimes planned, structured, repeated, and is intended to improve your physical fitness or to keep you fit. We are asking about physical activity, which not only includes exercise, but also other activities that involve bodily movement and are done as part of playing, working, active transportation, house chores and recreational activities. Sedentary behaviors are times when you are awake; when you are sitting, reclining, or lying down; and when your body uses very little energy. For adults, examples of sedentary behaviors include using electronic devices (e.g., television, computer, laptop, tablet, phone) while sitting, reclining or lying; reading, writing, or talking while sitting; sitting in a bus, car, or train.”

The definition of physical activity was meant to conform to how the World Health Organization defines physical activity. After the page with definitions, participants were presented with the 21-item physical activity and sedentary behavior identity scale on 3 separate screens. On each screen, participants were presented with 7 items designed to measure physical activity and sedentary behavior identity, plus 1 item that directed participants to pick a specific response option as an attention checker. Each of these 3 screens was timed, and the item order within each screen was randomized. After completing the identity scale, participants were asked to complete the IPAQ-SF. Participants who completed all parts of the survey were provided with a completion code to submit on their HIT for approval. MTurkers whose HIT was approved received US $3 compensation via Amazon MTurk.

#### Study 2

The study sample included participants of a study that involved collecting multiple physical activity assessments over the course of a week. Data from study 2 were used to evaluate the test-retest reliability of the physical activity and sedentary behavior identity scale. A total of 359 participants were recruited through the Understanding America Study (UAS) panel between January 2023 and July 2023. The UAS is a probability-based internet panel that longitudinally tracks a sample of approximately 10,000 US residents [15]. UAS panelists are recruited through address-based sampling. For potential panelists without internet access, the UAS provides a tablet and broadband access to ensure that the panelist pool achieves coverage in populations typically underrepresented in opt-in or volunteer online panels. A stratified random sample of the full UAS panel based on gender, race, and age was invited to participate in the study. There were 1363 panelists over the age of 18 years who responded to an invitation to participate in the study. Among these, 342 panelists did not meet the eligibility criteria for study participation, including having visual or audio impairment (n=209), requiring an assistive-mobility device (n=60), having no Wi-Fi access (n=26), having no stable access to email (n=32), working a night shift (n=75), being not fluent in English (n=12), being younger than 18 years (n=1), or being on bed rest (n=13). Among the 1021 eligible panelists, 407 (40% of those eligible) provided consent and started the study activity, and 359 (88.2% of those who consented) completed all the study-related activities. Participants of study 2 received US $10 for completing the surveys via a reloadable card that was provided to all the UAS panelists.

Study 2 participants completed the physical activity and sedentary behavior identity scale twice: at baseline and 7 days later. At 7 days after the baseline, study 2 participants also completed the IPAQ-SF. All instructions, definitions, and presentation of physical activity and sedentary behaviors, the identity scale, and the IPAQ-SF in study 2 were identical to those in study 1.

#### Data Handling

For both studies, we applied data cleaning procedures and criteria for removing outlying observations following the IPAQ-SF scoring guidelines recommended by the IPAQ Research Committee [16]. Accordingly, participants who met any of the following conditions were excluded from the analytic data set for both studies: (1) reported “Don’t know/Not sure” for the times spent walking, engaged in moderate physical activity, or engaged in vigorous physical activity and (2) the sum of daily times spent walking, engaged in moderate physical activity, and engaged in vigorous physical activity exceeded 960 minutes (16 hours; which was deemed unreasonably high assuming, on average, an individual had 8 hours of sleep duration per day). This yielded analytic samples of 848 (84.8%) of the 1000 study 1 participants who completed the study and 278 (77.4%) of the 359 study 2 participants who completed the study for study 1 and study 2, respectively. All the collected data were anonymous.

### Ethical Considerations

The procedures for both studies were approved by the University of Southern California Institutional Review Board (UP-21-00713) and Biomedical Research Alliance of New York (BRANY) Institutional Review Board (#22-183-1044). All participants provided informed consent before completing the study procedures.

### Data Analysis

Analyses to evaluate the factor structure underlying the responses to the physical activity and sedentary behavior identity items were conducted sequentially using data from study 1. Results from previous studies conducted using the original Exercise Identity Scale items suggested either 1 [8] or 2 [17] factors underlying the responses to the original scale, and we had added a set of items targeting sedentary behavior identity, which we expected to be indicators of 1 or 2 additional factors. Correspondingly, we examined exploratory factor analysis (EFA) models with 1 to 4 factors with oblique geomin rotation and compared models with increasing numbers of factors using likelihood-ratio tests. The preferred model was selected based on interpretability (high factor loadings >0.40 on conceptually interpretable item combinations with cross-loadings <0.40) and on model fit. Global model fit was evaluated using the chi-square goodness of fit test, comparative fit index (CFI; >0.95 for good model fit), Tucker-Lewis Index (TLI; >0.95 for a good fit), and root mean square error of approximation (RMSEA; <0.06 for a good fit) [18].

The EFA results in study 1 were used to inform the number and composition of factors in subsequent confirmatory factor analysis (CFA) models. CFA was used to evaluate the global fit of a measurement model without cross-loadings (ie, each item was allowed to load only on 1 factor). Items with substantial cross-loadings in EFA were excluded from the CFA. Because we aimed to generate brief scales with well-fitting measurement models, modification indices were examined to identify potentially problematic items that should be eliminated from the final models. The final (best-fitting) CFA model from study 1 was subsequently applied to the data collected in study 2 to evaluate whether the factor structure replicated across independent samples. The internal consistency reliability estimates of the resulting scale, descriptive statistics, and bivariate correlations among the subscales were examined using data from both studies 1 and 2. Convergent validity was assessed using the bivariate correlation between variables of the same constructs (eg, the correlation between physical activity identity scale and each of the 3 physical activity variables). Discriminant validity was assessed by comparing the correlation coefficient of the same construct (eg, the correlation between physical activity identity and physical activity behavior) with the correlation coefficient of a different construct (eg, the correlation between sedentary behavior identity and physical activity behavior) using tests for differences in dependent correlations [19] with data from study 1 and the week 2 data from study 2 when both IPAQ-SF and physical activity and sedentary behavior identity data were collected. The test-retest reliability of the resulting scale was examined using Pearson correlations and intraclass correlation coefficients using data from study 2. Descriptive statistics, internal consistency indices, and bivariate correlations were conducted using SAS 9.4 (SAS Institute). The factor analyses were conducted in Mplus version 8.7 [20] using maximum likelihood estimation. Comparisons of correlation coefficients were conducted using the online application developed by Lee and Preacher [21].

## Results

### Descriptive Statistics

Demographic characteristics of participants in studies 1 and 2 are presented in Table 1. On average, study 1 participants reported engaging in 177.2 (SD 266.1; range 0-270) minutes of vigorous physical activity, 325.84 (SD 436.33; range 0-2940) minutes of moderate physical activity, and 382.77 (SD 479.75; range 210-3360) minutes of walking during the past week. Study 1 participants reported an average daily sitting time during weekdays of 389.67 (SD 222.24; range 0-1230) minutes. Study 2 participants reported engaging in 101.4 (SD 190.7; range 0-1260) minutes of vigorous physical activity, 263.3 (SD 342.7; range 0-1260) minutes of moderate physical activity, and 431.7 (SD 408.6; range 0-1260) minutes of walking during the past week. Study 2 participants reported an average daily sitting time during weekdays of 448.6 (SD 236.9; range 30-1440) minutes.

**Table 1:** Participant demographic characteristics.

Characteristics			Study 1 (n=848)		Study 2 (n=278)
Female gender, n (%)			366 (43.2)		140 (50.4)
**Age group** **(years), n (%)**					
	18-39	493 (58.1)		83 (29.9)	
	40-59	285 (33.6)		114 (41)	
	60-79	70 (8.3)		75 (27)	
	≥80	0 (0)		6 (2.2)	
**Ethnicity, n (%)**					
	Non-Hispanic White	628 (74.1)		198 (71.2)	
	Hispanic White	72 (8.5)		27 (9.7)	
	Asian	52 (6.1)		15 (5.4)	
	Black or African American	61 (7.2)		17 (6.1)	
	American Indian or Alaska Native	3 (0.4)		2 (0.7)	
	Native Hawaiian or Pacific Islander	1 (0.1)		2 (0.7)	
	Multiracial	31 (3.6)		17 (6.1)	
**Education, n (%)**					
	High school degree or less	101 (11.9)		41 (14.8)	
	Some college: associate or no degree	217 (25.6)		78 (28.1)	
	Bachelor degree or higher	525 (61.9)		159 (57.2)	
	Prefer not to reply	5 (0.6)		0 (0)	
**Income** **(US $), n (%)**					
	<25,000	146 (17.2)		24 (8.6)	
	25,000 to <50,000	247 (29.1)		41 (14.8)	
	50,000 to <75,000	207 (24.4)		45 (16.2)	
	75,000 to <100,000	118 (13.9)		41 (14.8)	
	≥100,000	119 (14)		127 (45.7)	
	Prefer not to reply	11 (1.3)		0 (0)	
**Marital status, n (%)**					
	Married	405 (47.8)		179 (64.4)	
	Never married	355 (41.9)		59 (21.2)	
	Divorced, separated, or widowed	79 (9.3)		40 (14.4)	
	Prefer not to reply	9 (1.1)		0 (0)	
**Employment status, n (%)**					
	Employed (full-time, part-time, self-employed)	746 (88)		186 (66.9)	
	Student	8 (0.9)		0 (0)	
	Homemaker	26 (3.1)		0 (0)	
	Retired	26 (3.1)		47 (16.9)	
	Unemployed (out of work, not working by choice, unable to work)	33 (3.9)		44 (15.8)	
	Prefer not to reply	9 (1.1)		1 (0.4)	

### Factor Analysis

Using data from study 1, the initial EFA models suggested that retaining 1 or 2 factors resulted in poor model fit, even though the rotated solution of the 2-factor model was consistent with factors representing “physical activity identity” and “sedentary behavior identity” (see Table S2 in Multimedia Appendix 2). A model with 3 factors showed a near-acceptable fit with few cross-loadings (Table 2). The 3-factor model preserved the sedentary behavior identity factor, whereas items tapping physical activity identity loaded on two separate factors (see Table S2 in Multimedia Appendix 2). A 4-factor EFA model showed an acceptable fit, but the solution was difficult to interpret with many items loading substantially on multiple factors. Thus, the 3-factor model was retained (where 3 items with cross-loadings >0.40 were removed) and tested using CFA. Global fit indices in this CFA indicated near-acceptable fit (goodness of fit χ^2^_132_=1042.8, *P*<.001; CFI=0.94, TLI=0.93, RMSEA=0.084), but inspecting the modification indices suggested that the fit could be further improved by eliminating 6 additional items. After this reduction, the final model fit the data well (goodness of fit χ^2^_51_= 207, *P*<.001; CFI=0.98, TLI=0.98, RMSEA=0.056), and it was comprised of 4 items for each of the 3 factors. The factors were labeled “physical activity role identity,” “physical activity beliefs” (consistent with the labeling in [17], which found evidence for a similar factor structure), and “sedentary behavior identity.” Standardized loadings of the items in the final model are shown in Table 3.

When the final CFA from study 1 was applied to the data in study 2, the model showed acceptable fit in study 2 at baseline (goodness of fit χ^2^_51_=111.81, *P*<.001; CFI=0.98, TLI=0.98, RMSEA=0.058) and 1 week later (goodness of fit χ^2^_51_=178.00, *P*<.001; CFI=0.96, TLI=0.94, RMSEA=0.084).

**Table 2:** Exploratory factor analysis model fit using data from study 1.

Model	Parameters, n	χ^2^ (*df*)	CFI^a^	TLI^b^	RMSEA^c^
1-factor	63	5855.677 (189)	0.66	0.63	0.17
2-factor	83	1410.88 (169)	0.92	0.91	0.09
3-factor	102	774.81 (150)	0.96	0.95	0.07
4-factor	120	475.592 (132)	0.98	0.97	0.05

^a^CFI: comparative fit index.

^b^TLI: Tucker-Lewis index.

^c^RMSEA: root mean square error of approximation.

**Table 3:** Standardized loadings of the final factor solution using data from study 1.

Factors and included items		Standardized loading
**Factor 1: Physical activity role identity**		
	I consider myself to be a physically active person.	0.94
	Others see me as someone who is physically active regularly.	0.91
	I would describe myself as someone who is physically active.	0.94
	I would describe myself as someone who is more active than what’s typical for people like me.	0.83
**Factor 2: Physical activity beliefs**		
	I need to be physically active to feel good about myself.	0.79
	I have numerous goals related to physical activity.	0.81
	For me, being physically active means more than just performing physical activity.	0.64
	I would feel a real loss if I were not able to be physically active.	0.66
**Factor 3: Sedentary behavior identity**		
	I consider myself as a sedentary person.	0.86
	Others see me as a couch potato.	0.81
	When I am home, I want to sit, recline, or lie down more than anything else.	0.82
	I consider myself someone that sits (without standing) for long durations of time.	0.81

### Descriptive Statistics, Reliability Estimates, and Bivariate Correlations Among the Scale Scores and IPAQ-SF

Descriptive statistics of the 3 resulting scale scores are presented in Table 4. The 3 scale scores exhibited high internal consistency. For study 1 participants, the Cronbach α for physical activity role identity was 0.95, for physical activity belief was 0.81, and for sedentary behavior role identity was 0.89. Similar internal consistencies for the 3 subscales were observed both at baseline (Cronbach α=0.94 for physical activity role identity, Cronbach α=0.81 for physical activity belief, and Cronbach α=0.84 for sedentary behavior role identity) and 1 week later (ie, Cronbach α=0.94 for physical activity role identity, Cronbach α=0.79 for physical activity belief, and Cronbach α=0.84 for sedentary behavior role identity) among study 2 participants. The test-retest reliability (Pearson correlation) was 0.87 for physical activity role identity, 0.75 for physical activity belief, and 0.85 for sedentary behavior role identity and, when applying intraclass correlation coefficients, was 0.85 for physical activity role identity, 0.75 for physical activity belief, and 0.84 for sedentary behavior role identity.

For both studies, the scale scores for factor 1 (physical activity role identity) and factor 2 (physical activity belief) were strongly positively correlated (Pearson correlation coefficients [*r*] of 0.76 for study 1 and 0.69 for study 2). Factor 1 was moderately to strongly negatively correlated with factor 3 (sedentary behavior role identity, *r*=–0.58 for study 1 and *r*=–0.73 for study 2). Factor 2 was moderately negatively associated with factor 3 (*r*=–0.46 for both studies). As shown in Table 5, both physical activity role identity and physical activity belief scores were positively correlated with IPQA-SF–assessed times spent walking, engaged in moderate physical activity, and engaged in vigorous physical activity, and both were negatively correlated with time spent sitting, with correlations ranging from *r*=0.16 to *r*=0.41 in absolute magnitude. The sedentary behavior identity scale score was positively associated with time spent sitting and negatively associated with times spent walking, engaged in moderate physical activity, and engaged in vigorous physical activity, with correlations ranging from *r*=0.16 to *r*=0.38 in absolute magnitude.

**Table 4:** Descriptive statistics of the 3 scale scores and other physical activity (PA) and sedentary behavior (SB) measures.

Variables	Study 1, mean (SD)	Study 2, mean (SD)
Factor 1: PA role identity	4.59 (1.70)	(1.72)
Factor 2: PA belief	4.97 (1.36)	(1.36)
Factor 3: SB role identity	3.71 (1.66)	(1.53)
Weekly time spent in vigorous PA	177.23 (266.06)	101.4 (190.7)
Weekly time spent in moderate PA	325.84 (436.33)	263.3 (342.7)
Weekly time spent walking	382.77 (479.75)	431.7 (408.6)
Weekday daily time spent sitting	389.67 (222.24)	448.6 (236.9)

**Table 5:** Pearson correlations among the identity scale scores and self-reported physical activity (PA) and sedentary behavior (SB) using data from study 1 and data from the 1-week follow-up visit in study 2.

Variables		Study 1			Study 2		
		Factor 1: PA role identity	Factor 2: PA belief	Factor 3: SB role identity^a^	Factor 1: PA role identity	Factor 2: PA belief	Factor 3: SB role identity^a^
**Factor 1: PA role identity**							
	*r*	—^b^	0.76	–0.58	—	0.69	–0.73
	*P* value	—	<.001	<.001	—	<.001	<.001
**Factor 2: PA belief**							
	*r*	0.76	—	–0.46	0.69	—	–0.46
	*P* value	<.001	—	<.001	<.001	—	<.001
**Factor 3: SB role identity**							
	r	–0.58	–0.46	—	–0.73	–0.46	—
	*P* value	<.001	<.001	—	<.001	<.001	—
**Weekly time spent in vigorous PA**							
	*r*	0.40^c,d^	0.28 ^c,e^	–0.16^d,e^	0.41^d^	0.34	–0.33^d^
	*P* value	<.001	<.001	<.001	<.001	<.001	<.001
**Weekly time spent in moderate PA**							
	*r*	0.27^c,d^	0.21^c^	–0.20^d^	0.37^d^	0.34	–0.28^d^
	*P* value	<.001	<.001	<.001	<.001	<.001	<.001
**Weekly time spent walking**							
	*r*	0.26^c^	0.16^c,d^	–0.23^d^	0.27^c^	0.17^c^	–0.30
	*P* value	<.001	<.001	<.001	<.001	<.001	<.001
**Weekday daily time spent sitting**							
	*r*	–0.48^c,d^	–0.34^c^	0.38^e^	–0.33^c^	–0.23^c^	0.38
	*P* value	<.001	<.001	<.001	<.001	<.001	<.001

^a^Because SB role identity was negatively correlated with PA role identity and PA belief, correlations were compared after reverse scoring SB role identity.

^b^Not applicable.

^c,d,e^Within the columns of each study, the correlation coefficients in a row with the same superscripts differed significantly from each other in absolute magnitude (*P*<.05).

### Discriminant Validity

Several significant differences were evident when comparing the 3 identity scales on the magnitude of their correlations with self-reported physical activity levels (see Table 5). Comparing physical activity role identity with physical activity belief, the physical activity role identity scale showed significantly larger correlations with times engaged in vigorous and moderate activities (in study 1) as well as with times walking and sitting (in studies 1 and 2) compared with the physical activity belief scale. Comparing physical activity role identity with sedentary behavior role identity, the physical activity role identity scale showed significantly larger correlations with times engaged in vigorous and moderate activities (in studies 1 and 2) compared with the sedentary behavior role identity scale; contrary to expectation, the physical activity role identity scale also showed a larger correlation with time spent sitting (in study 1) compared with the sedentary behavior role identity scale. Comparing physical activity belief with sedentary behavior role identity, the physical activity belief scale showed a larger correlation with time engaged in vigorous activities (study 1) and a lower correlation with time spent walking (study 1) compared with the sedentary behavior role identity scale.

## Discussion

The purpose of these studies was to develop a revised physical activity and sedentary behavior identity scale, followed by examining the psychometric properties, convergent and discriminant validity, and test-retest reliability of the scale. We found evidence for 3 factors underlying the items, with 2 factors representing distinguishable facets of physical activity–related identity and 1 representing sedentary behavior role identity. Scale scores derived for all 3 factors were internally consistent. The scales presented in these studies also exhibited criterion validity, such that individuals who scored high on the physical activity role identity and physical activity belief subscales also reported spending more time in physical activity and less time sitting, while those who scored high on sedentary behavior role identity reported more time sitting and less time engaged in physical activity.

In prior research, Wilson and Muon [17] identified 2 factors underlying the Exercise Identity Scale, and the physical activity role identity factor and physical activity belief factor identified in this study are in line with this 2-factor model. Similar to results by Wilson and Muon [17], this study also observed that, although both of these identity scales were positively correlated with self-reported time spent in physical activity, the correlation was consistently stronger for physical activity role identity than for physical activity belief.

Findings from this study extend the existing literature in at least two ways. First, the study extends evidence for the utility of the physical activity identity scale [12] to a wider age range. Like the results reported by Strachan et al [12], this study also found a moderate positive correlation between physical activity identity and self-reported moderate and vigorous physical activity. This result may further suggest that physical activity identity is positively associated with physical activity behavior in adults in general, rather than just within the older adult population. Second, this study provided evidence that sedentary behavior identity can be distinguished from physical activity identity; sedentary behavior identity emerged as a separate factor in factor-analysis models, and it was positively associated with sedentary behavior but negatively associated with physical activity behavior. Although preliminary, these results support the potential validity of the sedentary behavior identity scale, which might be used to help understand factors that contribute to prolonged engagement in sedentary behavior and potentially serve as a target for behavior change.

Results of this study offer initial evidence on the psychometric properties of the physical activity and sedentary behavior identity scale. However, there are notable limitations of this study. Although participants in this study were adults from a wide age range, they were recruited from online participants and may be different from the general public. For example, workers from Amazon MTurk may differ from the general population in various ways (eg, younger, better educated [22], and lower life satisfaction [23]). Similarly, although the full UAS panel is a probability sample that represents the general US population, the panelists that were included in this study may differ from the general public; for example, over 40% of the panelists included in this study were from higher-income households. Therefore, future studies using samples from other age groups, ethnic composition, and income levels could potentially expand the current understanding of the characteristics of the physical activity and sedentary behavior identity scale. Additionally, evidence regarding the health effects of physical activity with different intentionality (eg, lifestyle-embedded physical activity, occupation-related physical activity) has started to emerge [24,25]. The mechanisms and correlates of physical activity may differ depending on the type of intentionality, and whether the utility of the revised physical activity and sedentary identity scale generalizes to physical activity of all intentionality types remains unexamined.

In conclusion, these studies provide initial empirical evidence on the reliability and validity of the physical activity and sedentary identity scales for adult participants across the age spectrum using 2 independent studies. Emerging evidence has demonstrated the utility of identity in promoting physical activity engagement [7]. The scales described in this study could provide a useful measurement approach for identities related to physical activity and sedentary behavior to enrich the current understanding of the role of physical activity and sedentary behavior identity in the field of physical activity promotion and sedentary behavior reduction efforts.

## Appendix

Multimedia Appendix 1 Candidate items for physical activity and sedentary behavior identity.

Multimedia Appendix 2 Geomin rotated standardized factor loadings from exploratory factor analysis with 2 and 3 factors.

## Abbreviations

BRANY: Biomedical Research Alliance of New York
CFA: confirmatory factor analysis
CFI: comparative fit index
EFA: exploratory factor analysis
HIT: human intelligence task
IPAQ-SF: International Physical Activity Questionnaire short form
MTurk: Mechanical Turk
RMSEA: root mean square error of approximation
TLI: Tucker-Lewis index
UAS: Understanding America Study
