# Supplementary material for: Evaluating the Psychometric Properties of a Physical Activity and Sedentary Behavior Identity Scale: Survey Study With Two Independent Samples of Adults in the United States
Source: JMIR Form Res. 2024 Oct 24;8:e59950. doi: 10.2196/59950 (PMC11544334; doi:10.2196/59950)
Supplement: Multimedia Appendix 1 [file formative_v8i1e59950_app1.docx]

Table S1.

List of candidate items for physical activity and sedentary behavior identity

Candidate for physical activity identity scale

1. I consider myself to be a physically active person.
2. When I describe myself to others, I usually include my involvement in physical activity.
3. Being physically active is a central factor to my self-concept.
4. I need to be physically active to feel good about myself.
5. Others see me as someone who is physically active regularly.
6. I have numerous goals related to physical activity.
7. For me, being physically active means more than just performing physical activity.
8. I would feel a real loss if I were not able to be physically active.
9. Physical activity is something I think about often.
10. I would describe myself as someone who is physically active.
11. During my free time, I enjoy activities that allow me to get up and move more than most other activities.
12. I would describe myself as someone who is more active than what’s typical for people like me.

Candidates for sedentary behavior identity scale

1. I consider myself as a sedentary person.
2. When I describe myself to others, I usually include my involvements in activities that are sedentary.
3. I would be disappointed if my activities were limited to those that required me to lie down, sit, or recline.
4. I often think about being sedentary.
5. Others see me as a couch potato.
6. I would describe myself as someone that sits more than is typical for people like me.
7. I enjoy being sedentary during my free (or leisure) time.
8. When I am home, I want to sit, recline, or lie down more than anything else.
9. I consider myself someone that sits (without standing) for long durations of time.
