# Supplementary material for: Evaluating the Psychometric Properties of a Physical Activity and Sedentary Behavior Identity Scale: Survey Study With Two Independent Samples of Adults in the United States
Source: JMIR Form Res. 2024 Oct 24;8:e59950. doi: 10.2196/59950 (PMC11544334; doi:10.2196/59950)
Supplement: Multimedia Appendix 2 [file formative_v8i1e59950_app2.docx]

Table S2. Geomin rotated standardized factor loadings from exploratory factor analysis with 2 and 3 factors

|  | 2-factor solution | | 3-factor solution | | |
| --- | --- | --- | --- | --- | --- |
|  | Factor 1 | Factor 2 | Factor 1 | Factor 2 | Factor 3 |
| I consider myself to be a physically active person. | 0.84* | -0.16* | 0.94* | -0.03 | -0.08* |
| When I describe myself to others, I usually include my involvement in physical activity. | 0.89* | 0.17* | 0.61* | 0.28* | 0.18* |
| Being physically active is a central factor to my self-concept. | 0.86* | 0.00 | 0.48* | 0.44* | 0.01 |
| I need to be physically active to feel good about myself. | 0.75* | 0.01 | 0.26* | 0.56* | -0.01 |
| Others see me as someone who is physically active regularly. | 0.86* | -0.09* | 0.87* | 0.05 | -0.02 |
| I would describe myself as someone who is physically active. | 0.83* | -0.17* | 0.90* | 0.01 | -0.09* |
| I have numerous goals related to physical activity. | 0.78* | 0.02 | 0.31* | 0.54* | 0.00 |
| For me, being physically active means more than just performing physical activity. | 0.63* | 0.03 | 0.16* | 0.53* | 0.00 |
| I would feel a real loss if I were not able to be physically active. | 0.51* | -0.18* | 0.10 | 0.53* | -0.19* |
| Physical activity is something I think about often. | 0.74* | 0.13* | 0.01 | 0.80* | 0.07* |
| During my free time, I enjoy activities that allow me to get up and move more than most other activities. | 0.73* | -0.11* | 0.41* | 0.41* | -0.10* |
| I would describe myself as someone who is more active than what’s typical for people like me. | 0.84* | -0.02 | 0.74* | 0.14* | 0.02 |
| I consider myself as a sedentary person. | -0.07* | 0.84* | -0.30* | 0.00 | 0.74* |
| When I describe myself to others, I usually include my involvement in activities that are sedentary. | 0.24* | 0.69* | 0.02 | 0.05 | 0.63* |
| Others see me as a couch potato. | -0.01 | 0.81* | -0.25* | 0.02 | 0.72* |
| I would be disappointed if my activities were limited to those that required me to lie down, sit, or recline. | -0.38* | 0.20* | -0.05 | -0.44* | 0.21* |
| I often think about being sedentary. | 0.32* | 0.86* | -0.08 | 0.19* | 0.77* |
| I enjoy being sedentary during my free (or leisure) time. | -0.01 | 0.69* | 0.10* | -0.32* | 0.67* |
| I would describe myself as someone that sits more than is typical for people like me. | 0.03 | 0.89* | -0.20* | -0.01 | 0.80* |
| When I am home, I want to sit, recline, or lie down more than anything else. | -0.01 | 0.80* | 0.01 | -0.26* | 0.76* |
| I consider myself someone that sits (without standing) for long durations of time. | -0.07* | 0.78* | -0.23* | -0.06 | 0.70* |

*Note*: **p* <. 05
